# Acupuncture-assisted therapy for prolonged disorders of consciousness: study protocol for a randomized, conventional-controlled, assessor-and-statistician-blinded trial

**DOI:** 10.3389/fneur.2024.1334483

**Published:** 2024-09-03

**Authors:** Na Zhi, Ning Sun, Pan Huang, Li-yuan Yang, Cai-xia Guo, Jing Xiong, Yi-wei Liu, Hong Zhang

**Affiliations:** ^1^Acupuncture and Tuina School, Chengdu University of Traditional Chinese Medicine, Chengdu, Sichuan, China; ^2^Rehabilitation Medicine Center and Institute of Rehabilitation Medicine, West China Hospital, Sichuan University, Chengdu, China; ^3^Rehabilitation Medicine Department, West China Tianfu Hospital, Sichuan University, Chengdu, China

**Keywords:** acupuncture, prolonged disorders of consciousness, randomized controlled trial, effectiveness and safety, study protocol

## Abstract

**Background:**

Acupuncture is a promising non-pharmaceutical complementary therapy in treating prolonged Disorders of consciousness (pDOC), but solid evidence to support its effectiveness and safety is still lacking. Thus, the purpose of this study is to investigate the efficacy and safety of acupuncture-assisted therapy for pDOC patients.

**Methods:**

A single-center, prospective, randomized, conventional-controlled, assessor-and-statistician-blinded trial has been designed and is being conducted at West China Hospital of Sichuan University. A total of 110 participants will be randomly assigned to the experimental group and the control group in a 1:1 allocation ratio and evaluated using Coma Recovery Scale-Revised (CRS-R) at 8 a.m., 12 p.m., and 4 p.m. on 2 consecutive days before enrollment to determine the consciousness level. The experimental group will receive acupuncture combined with conventional treatment, while the control group will receive only conventional treatment during the trial observation period. The treatment duration of both groups will be 20 days. Among them, the frequency of acupuncture-assisted therapy is once a day, with 10 consecutive sessions followed by a day’s rest for a total of 24 days. Data will be collected separately during baseline and after the final treatment. For data analysis, both Full Analysis Set (FAS) and Per Protocol Set (PPS) principles will be performed together by applying SPSS 27.0 software. The primary outcome measures are the changes of CRS-R before and after treatment, while the secondary outcome measures are the changes of Full Outline of Unresponsiveness Scale (FOUR), the changes of Nociception Coma Scale-Revised (NCS-R), the changes of Disability Rating Scale (DRS), the changes of Mismatch Negativity (MMN) and P300 before and after treatment, respectively.

**Discussion:**

This trial aims to rationally assess the consciousness level from multiple 2 perspectives through subjective evaluation and objective detection by selecting several standardized clinical scales combined with Event-Related Potential (ERP) detection technology. In this way, we will be able to reduce the subjectivity of consciousness assessment and objectively evaluate the clinical efficacy of acupuncture-assisted therapy for pDOC. The study, if proven to be effective and safe enough, will provide a favorable evidence to guide medical decision-making choices and future researches.

**Clinical trial registration:**

https://www.chictr.org.cn/, identifier ChiCTR2300076180.

## 1 Introduction

Disorders of consciousness (DOC) is a persistent state of loss of consciousness usually caused by structural damage or dysfunction of the neural systems that regulate wakefulness and awareness (1, 2). The main mechanism is related to brain cell metabolic malfunction attributed to cerebral ischemia, hypoxia, insufficient glucose supply, and abnormalities in enzyme metabolism, which leads to impaired reticular function and low brain function (3). When this state lasts longer than 28 days, these patients will be diagnosed with prolonged disorders of consciousness (pDOC) (4), which mainly include vegetative state (*VS*)/unresponsive wakefulness syndrome (UWS), and minimally conscious state (MCS) (5). Patients with *VS*/UWS retain the sleep–wake cycle, can open their eyes voluntarily, and remain awake, but are unable to perceive and respond accordingly to their own or environmental stimuli (6). In contrast, patients with MCS who also retain the sleep–wake cycle can show minimal but definite signs of awareness of themselves or the environment, which can include eye movements that track objects, show purposeful responses to stimuli, or cannot be interpreted as purely reflexive movements (2).

It has been reported that the prevalence of pDOC patients in the United States approaches 100,000–300,000 (7). Meanwhile, the prevalence of pDOC patients in Europe ranges from 0.2 to 6.1 cases per 100,000 individuals (8). Although there is no reliable data in China in terms of prevalence for pDOC, it is undeniable that the number of pDOC patients is increasing with the improvement of medical resuscitation techniques (9). Due to pDOC patients need long-term and continuous medical expenses as a support for their lives, which creates a severe burden and pressure on both families and society. The main goal of rehabilitation for pDOC patients is to improve their consciousness level and functional recovery. Currently, owing to the limited number of available therapies and their effectiveness in promoting wakefulness, it is meaningful to find a safe and effective long-term adjuvant therapy for pDOC patients.

Acupuncture, as a specialty therapy of Traditional Chinese Medicine (TCM) with a history of thousands of years, has been widely used to treat various ailments as a safe and effective therapy (10, 11). Preliminary studies have indicated that acupuncture not only promotes the recovery of consciousness, speech, and limb functions, reducing the disease duration but also improves the long-term quality of life for DOC patients, effectively reducing mortality and disability (12–14). Meanwhile, the efficacy of acupuncture-assisted therapy may be related to the mechanisms that acupuncture enhances blood supply and oxygenation in the lesion area, reduces the occurrence of edema developmental changes in the necrotic area, and rescues some neurons on the verge of loss of function, thus further promoting self-repairing of the damaged brain tissues and reconstruction of neural networks (15–17). To date, however, it appears from the results of our literature study that there have been no RCTs conducted on pDOC alone. Compared with DOC patients, pDOC patients suffer from the effects of more severe brain damage, more complications, and greater difficulty in promoting awakening. Consequently, a first investigation of the efficacy and safety of acupuncture-assisted treatment of pDOC is necessary. In addition, acupuncture has fewer side effects and low cost, which greatly improves the usefulness of acupuncture. Therefore, acupuncture may be a better complementary therapy for pDOC patients.

At present, behavioral assessment, based on patients’ motor and cognitive behaviors, is the main modality used in clinical practice to evaluate the consciousness level of pDOC patients. However, the misdiagnosis rate for this method of assessing consciousness state for patients with pDOC through the use of scale scores has been high, at approximately 40% (18, 19). In order to minimize the misdiagnosis rate and to objectively reflect the clinical efficacy of acupuncture-assisted therapy for pDOC. In this study, we selected several standardized clinical scales closely related to the consciousness level, and combined them with indicators for the detection of Event-Related Potentials (ERP), which together serve as indicators of efficacy evaluation, aiming to reasonably judge the clinical efficacy of acupuncture-assisted treatment of pDOC from the perspectives of subjective assessment and objective detection.

## 2 Methods and analyses

### 2.1 Design and setting

**Figure 1:**
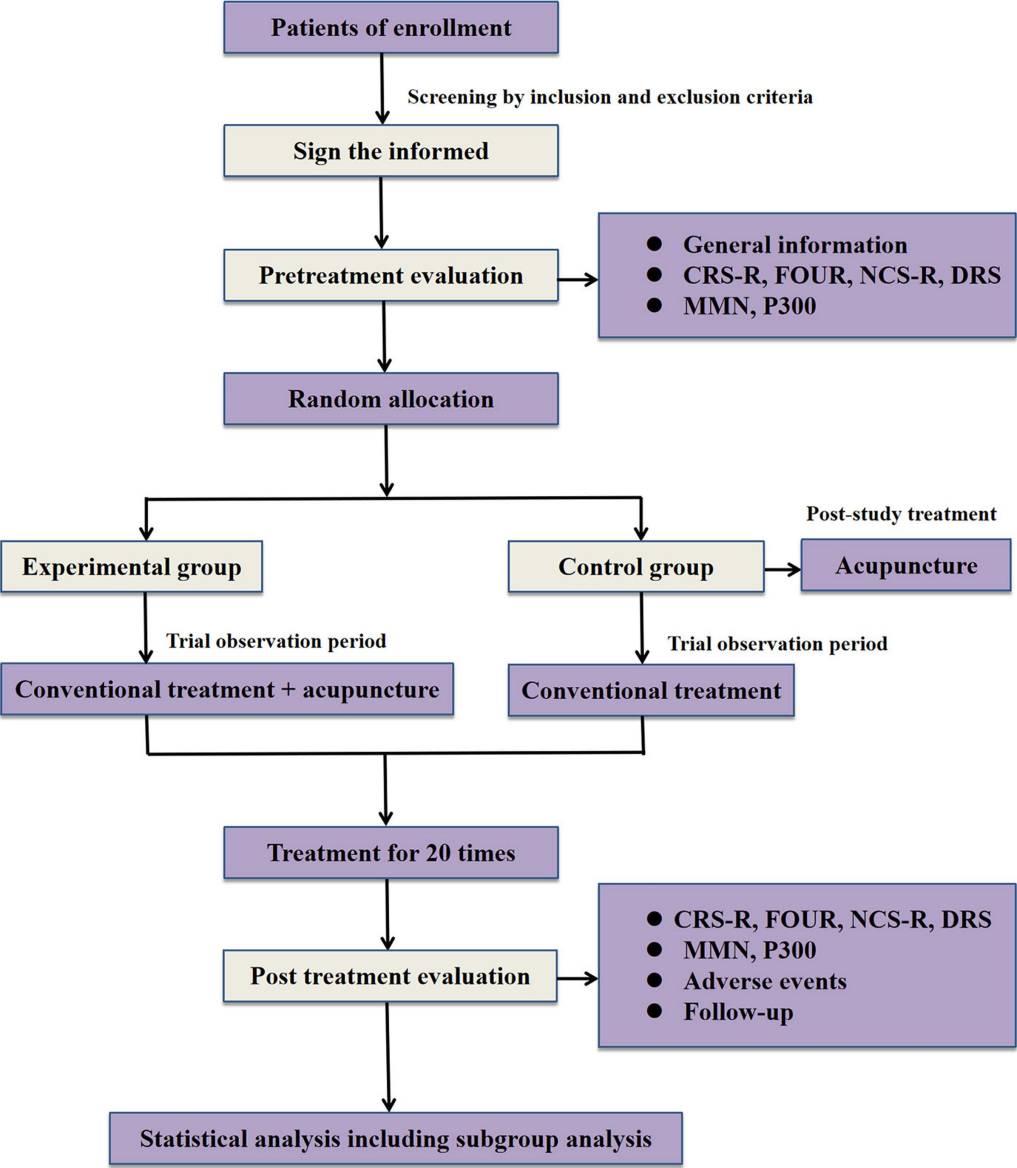
Experimental flow chart.

A single-center, prospective, randomized, conventional-controlled, assessor-and-statistician-blinded trial is being performed at West China Hospital of Sichuan University (Sichuan, China). A total of 110 eligible participants will be randomly assigned to the experimental group and the control group in a 1:1 allocation ratio. Acupuncture combined with conventional treatment will be compared to conventional treatment in patients with pDOC. The study was approved by the ethics committee of West China Hospital of Sichuan University under the ethical approval number 2022 review (1622), and registration was completed in the China clinical trial registry under the registration number ChiCTR2300076180. Also, this trial is implemented strictly conforms to Standard Protocol Items, such as Recommendations for Interventional Trials (SPIRIT) 2013 statement (20) and the Standards for Reporting Interventions in Controlled Trials of Acupuncture (STRICTA) (21). The process and details of this study will be demonstrated in Figure 1 and Table 1.

**Table 1:** Schedule of enrolment, interventions, and assessments.

**Time point**	**Study period**				
	**Screening**	**Baseline**	**Intervention**	**Post-treatment**	**Follow up**
**Enrollment**					
Eligibility screen	√	√			
Informed consent	√				
Medical history		√			
Random allocation		√			
Demographic data		√			
**Intervention**					
Conventional treatment + acupuncture (*n* = 55)			√		
Conventional treatment (*n* = 55)			√		
**Assessments**					
CRS-R		√		√	
FOUR		√		√	
NCS-R		√		√	
DRS		√		√	
MMN		√		√	
P300		√		√	
**Others**					
Adverse events			√	√	
Where pDOC patients go after being discharged from the hospital					√

CRS-R, the Coma Recovery Scale-Revised; FOUR, the Full Outline of Unresponsiveness Scale; NCS-R, the Nociception Coma Scale-Revised; DRS, the Disability Rating Scale; MMN, the Mismatch Negativity; pDOC, prolonged Disorders of Consciousness.

### 2.2 Patients

#### 2.2.1 Recruitment strategies

This trial will be advertised for recruitment between January 2023 and October 2024 via WeChat, the hospital’s official website, and the outpatient waiting hall. Subjects with pDOC were required to participate in our trial only after their family members and (or) legal representatives were informed, consented and signed an informed consent form. Meanwhile, they will be informed that patients with pDOC are free to withdraw from this trial at any time and that there will be no negative impact on their future treatment. Additionally, all personal information about the patient will be well protected and not casually disclosed.

##### 2.2.1.1 Diagnostic criteria

Based on the 2020 edition of the European guidelines for the diagnosis of coma and other DOC published by the European Academy of Neurology and the 2018 edition of the American practice guidelines for DOC published jointly by the American Academy of Neurology and the American academy of rehabilitation medicine (18, 22), we can know that patients with DOC more than 28 days will be diagnosed as pDOC.

##### 2.2.1.2 Inclusion criteria

Patients with pDOC meeting all of the following criteria will be enrolled in this trial. Otherwise, they will be excluded.

1. Patients who met the above diagnostic criteria for pDOC will be included only in the *VS*/UWS and MCS stages;
2. Those who have a first sudden onset of impaired consciousness with a clear etiology and a complete case history;
3. No significant hydrocephalus and no severe cerebral atrophy, 28 days < disease duration ≤6 months;
4. Age is between 18 and 80 years old and gender is not limited;
5. Those without significant cognitive impairment, hearing impairment, or visual impairment before onset;
6. Those who were able to communicate accurately in Chinese before the onset of the disease and who had no significant neurologic or psychiatric history;
7. Those with stable medical conditions, relatively stable vital signs, and not enrolled in another study within the last 3 months;
8. Informed consent was obtained from the subject’s legal representative, and the subject’s legal representative voluntarily cooperated and signed the informed consent form.

##### 2.2.1.3 Exclusion criteria

Patients with pDOC meeting any one of the following criteria will be excluded from our study.

1. Those with a combination of intracranial tumors, episodes of intracranial infections, or brain death and/or other occupying diseases that significantly affect the results of the study;
2. Those with a history of drug use prior to illness;
3. Those with deep vein thrombosis;
4. Those with severe endocrine-metabolic disorders or with persistent status epilepticus;
5. Pregnancy, combined multiple trauma, limb fractures, or large skin defects.

##### 2.2.1.4 Criteria for termination

Included pDOC patients meeting any one of the following criteria will be terminated from our trial.

1. Those whose vital signs were unstable due to changes in their condition during the observation period or who were advised by the managing physician that they could not continue to participate in this clinical study;
2. Those who require reoperation or die during observation;
3. Those who had a serious adverse reaction during the observation period and should not continue to participate in the study;
4. The legal representative does not cooperate with the study and repeated explanations by the clinician are not effective.

##### 2.2.1.5 Criteria for withdrawal, dropout, and removal

Included pDOC patients meeting any one of the following criteria will be withdrew, dropped out, and removed from our study.

1. Patients who did not meet the inclusion criteria and were included in error;
2. Subjects with poor compliance by legal representatives who withdrew on their own during the study;
3. Combined use of treatments prohibited by this program, or those who change treatments midway on their own;
4. Those who are unfit to continue in the study due to major changes in the family.

##### 2.2.1.6 Handling of exclusion and dropout cases

For pDOC patients who are kicked out and dislodged, we will take the following.

corresponding measures as appropriate to the situation to handle them appropriately.

1. In cases of dislodgement, the supervising physician should contact the subject’s family or legal representative by visiting the home, scheduling a phone call, or sending an email, as much as possible, to inquire about the cause of the dislodgement, to keep a detailed record of when the last treatment was given, and to complete as many of the evaluation items as he or she is able to;
2. If a person withdraws due to adverse reactions or ineffective treatment, the supervising physician shall take appropriate measures to deal with the subject according to his/her actual situation;
3. Complete the Case Report Form (CRF) “Treatment Completion Summary” and “Clinical Trial Completion”;
4. Once a pDOC patient is enrolled for observation, detailed records should be kept regardless of subsequent diagnosis and completeness of treatment;
5. The researcher should keep a detailed record of the reason and time of withdrawal from the study;
6. All rejected and dislodged cases were analyzed in the Full Analysis Set (FAS) at the end of the trial.

##### 2.2.1.7 Supplementary explanation

To fully consider the safety of the subjects and the real reliability of the data in the course of this trial, this study only collected relevant data from subjects who met the requirements of the ERP technology test, and the rest of the participants only underwent clinical evaluation and treatment.

ERP technology testing requirements: ① subjects quiet, not agitated, and able to complete the test; ② skull intact, and no other factors affected.

### 2.3 Intervention

#### 2.3.1 Conventional treatment in both groups

This study was divided into two groups, i.e., control group and experimental group. Subjects in both groups who participated in this trial will receive conventional clinical treatments with pDOC, including rehydration, anti-infection, seizure prevention, neurotrophic, acid-suppressing and gastric protection, mechanically assisted sputum evacuation, medication to resolve sputum, hyperbaric oxygen combined with transcranial direct current stimulation (tDCS) to promote awakening, physical rehabilitation, nutritional support, and other symptomatic treatments. Details of conventional treatment will be recorded carefully on the CRF to which the patient belongs, noting any change and the reasons promptly. The formulation of conventional treatment plans is mainly based on the “Chinese Expert Consensus on the Diagnosis and Treatment of pDOC” (23), closely related to the patient’s clinical condition, combined with the recommendations and opinions of experts in the field, and referred to the clinical pathway medication guidance of West China Hospital of Sichuan University.

The following are the details of the clinical conventional treatment plan for pDOC patients: Renen 400 mL via gastric tube once a day; Ambroxol hydrochloride injection 30 mg intravenous drip combined with inhalation of acetylcysteine solution nebulized inhalation once a day; piperacillin sodium tazobactam sodium for injection 4.5 g IV drip every 8 h; omeprazole sodium 80 mg, 0.9% saline diluted in 100 mL of IV drip, once a day; Depakene 5 mg/kg dissolved in 100 mL saline IV drip within 1 h, every 8 h; Odekin 0.8 g IV drip, once a day. Hyperbaric oxygen therapy: Set the pressure of the hyperbaric oxygen chamber to 0.2 MPa, slowly and continuously pressurize for 20 min, wear a mask to inhale oxygen for 30–40 min per time, a total of two times, with a 10 min interval between each time, during which the air inside the chamber is sucked. After the two oxygen breaths are completed, slowly reduce the pressure and exit the chamber, once a day. tDCS therapy: The dorsolateral left prefrontal lobe was chosen as the anode, and the dorsolateral right prefrontal lobe was chosen as the cathode, with a stimulation intensity of 1 ~ 2 mA and a duration of 20 min, once a day, and with enhanced limb rehabilitation, bed standing and other treatments.

#### 2.3.2 The experimental group

**Figure 2:**
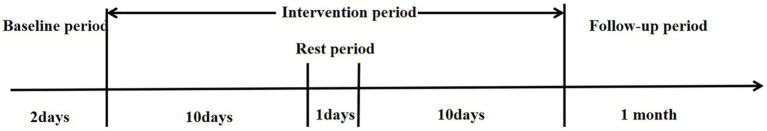
Time allocation chart.

In the 20-day treatment phase, participants that allocated to the experimental group will receive acupuncture treatment in addition to conventional treatment. The frequency of acupuncture treatment will be once a day, with 1 day of rest after 10 consecutive treatments, for a total of 20 treatments. The distribution of observation time regarding the participation of pDOC patients in our trial was shown in Figure 2. The selection of acupoints in this study was mainly based on the results of data mining and consisted of acupoints that were selected a high number of times in the current application of clinical acupuncture-assisted therapy for DOC patients (24). Meanwhile, all acupoints will be localized according to the Chinese standard “Names and Localization of Meridian Points” (GB/T12346-2021), and the acupuncture manipulation techniques are mainly based on the “International Technical Operation Standards for Traditional Chinese Medicine - Xingnao Kaiqiao Acupuncture Method for the Treatment of Stroke,” which was released and implemented by the World Federation of Traditional Chinese Medicine Societies on December 16, 2021, as the main guideline (25, 26).

Among all selected acupoints, Shuigou (GV26) and bilateral Neiguan (PC6) were the main points, and the remaining acupoints were all supporting points, including Baihui (GV20), double Fengchi (GB20), double Jiquan (HT1), double Chize (LU5), double Hegu (LI4), double Laogong (PC8), double Houxi (SI3), double Zusanli (ST36), double Yinlingquan (SP9), double Sanyinjiao (SP6), double Taixi (KI3), double Yongquan (KI1), and double Taichong (LR3). The details of acupoint localization are shown in Table 2.

**Table 2:** The details of acupoint localization.

**Acupoints**	**Anatomical location**
Shuigou (GV26)	On the face, at the junction of the upper 1/3 and lower 2/3 of the manubrium sulcus.
Neiguan (PC6)	In the anterior region of the forearm, 2 inches above the transverse stripe on the distal part of the palmar side of the wrist, between the tendon of the palmaris longus and the tendon of the radial flexor carpi radialis.
Baihui (GV20)	On the head, 5 inches straight up from the center of the front hairline.
Fengchi (GB20)	In the posterior region of the neck, below the occipital bone, in the depression between the upper end of the sternocleidomastoid muscle and the upper end of the trapezius muscle.
Jiquan (HT1)	In the center of the axilla, where the axillary artery pulsates, the acupuncture point in this study was Lower Polar Spring, in the anterior region of the arm, on the line connecting the Polar Spring and the Shao Hai, 1 ~ 2 inches below the Polar Spring, avoiding the axillary hairs, and at the point where the muscles are plentiful.
Chize (LU5)	In the elbow region, on the transverse elbow stripe, in the radial depression of the biceps tendon.
Hegu (LI4)	On the back of the hand, between the 1st and 2nd metacarpals, at the midpoint of the radial side of the 2nd metacarpal.
Laogong (PC8)	In the metacarpal region, transverse to the proximal end of the 3rd metacarpophalangeal joint, favoring the 3rd metacarpal between the 2nd and 3rd metacarpals.
Houxi (SI3)	In the depression between the red and white flesh at the proximal end of the ulnar side of the 5th metacarpophalangeal joint of the hand.
Zusanli (ST36)	On the lateral side of the lower leg, 3 inches below the calf’s nose, 1 transverse finger outside the anterior crest of the tibia, on the line between the calf’s nose and Xiexi.
Yinlingquan (SP9)	On the medial side of the lower leg, in the depression between the lower edge of the medial tibial condyle and the medial border of the tibia.
Sanyinjiao (SP6)	On the medial side of the lower leg, 3 inches above the tip of the inner ankle, behind the medial border of the tibia.
Taixi (KI3)	In the ankle region, in the depression between the tip of the inner ankle and the Achilles tendon.
Yongquan (KI1)	On the sole of the foot, the center of the foot is most concave in the center of the foot when flexing the foot and curling the toes (when the webbing edge of the 2nd and 3rd toes on the sole of the foot meets the intersection of the anterior 1/3 and posterior 2/3 of the line connecting the heel of the foot).
Taichong (LR3)	On the dorsum of the foot, between the 1st and 2nd metatarsals, in the depression anterior to the union of the metatarsal bottoms, or where the arterial pulsation is palpable.

#### 2.3.3 The control group

At the end of the study observation, all participants in the control group will be entitled to 20 free acupuncture intervention treatments as in the experimental group, and if all of them cannot be completed during their first hospitalization, the subject’s entitlement will remain in effect for future hospitalizations at our institution until the 20th acupuncture treatment is completed.

#### 2.3.4 Acupuncture operation process

All acupuncture operators have at least 5 years of clinical experience in acupuncture therapy and have obtained the doctor’s qualification certificate. Their acupuncture manipulation has undergone unified standardized training and reached the qualification standard. During the acupuncture operation, the operator wears isolation clothing and strictly follows the principle of using a separate set of isolation clothing for each pDOC patient to avoid cross infection of drug-resistant bacteria among patients.

##### 2.3.4.1 Sterilize

Before applying the needles, the acupuncture operator brushes and cleans his/her hands with soapy water and then wipes them with a 75% alcohol cotton ball. Next, they used 75% alcohol cotton balls to disinfect the skin of the needling site in a circular manner from the acupoint area to the periphery.

##### 2.3.4.2 Needle insertion and needle travel

We choose the appropriate needle insertion technique based on the different injection sites, and strictly follow the acupuncture operation requirements corresponding to the acupoints to perform acupuncture techniques. The acupuncture operation is based on Shi’s twisting tonic and diarrhea technique, when twisting, small amplitude, high frequency (twisting amplitude less than 90°, frequency of 120 ~ 160 times/min) for the tonic method. On the contrary, large amplitude, low frequency (twisting amplitude of more than 180°, frequency of 60 ~ 90 times/min) for the diarrhea method (25, 26).

##### 2.3.4.3 Acupuncture manipulation

In the process of acupuncture manipulation, it is worth noting that when needling GV26, the needle tip should be obliquely inserted toward the nasal septum by 0.3–0.5 inches, and then acupuncture operations such as lifting and twisting should be performed. The intensity of stimulation should be measured by the patient’s tears or moist eyeballs. Next, when needling PC6, the tip of the needle should be perpendicular to the skin into the needle about 0.5-1inches, and then use the lifting and inserting twisting and diarrhea method, and continue to travel the needle for 1 min. Meanwhile, it should be emphasized that in addition to the need to cause twitching of the upper limbs when needling the HT1, it is also necessary to avoid its adjacent axillary artery as a means of avoiding acupuncture accidents, and needling the LU5, LU5 should be positioned by bending the elbow at an internal angle of 120°, and the operator should hold the wrist joint of the affected limb with his hand, stabbing 0.5 ~ 0.8 inches straight, using the lift-and-insert diarrhea method, to make the needle sensation pass from the elbow joint to the fingers or to manually externally rotate the hand, and to externally rotate the hand and twitch it for 3 times as a criterion. In addition, SP6 should be stabbed obliquely along the medial edge of the tibia at an angle of 45° to the skin, and the needle should be advanced about 0.5 to 1 inch so that the tip of the needle is stabbed deeply into the position of the original SP6, and then the lifting and inserting method of tonic is used to the extent that the affected limb twitches three times. GV20 should be stabbed backward flatly for 1 inch, and then perform flat tonic and flat diarrhea maneuvers, and twist rapidly for 1 min. When needling GB20, the needle tip should be directed towards the opposite side of the eyeball and stabbed directly for 1–1.5 inches. A small amplitude and high-frequency twisting and turning method should be applied for 1 min. Additionally, when needling LI4, for patients with finger dysfunction, the acupoint on the affected side should be taken, and then the needle should be inserted with one hand, and the needle tip should be stabbed obliquely in the direction of the San Jian (LI3) for 1 ~ 1.5 inches, and then the lifting and inserting and cathartic methods should be used, to the extent that the patient’s tightly clenched finger naturally stretches out or the index finger involuntarily jerks three times. Lift and insert the laxative method, to the extent that the patient’s thumb twitches 3 times; for patients without finger dysfunction, the bilateral LI4 is subjected to the acupuncture maneuver of flat tonic and flat laxative. When needling KI1, we used a combination of lifting, inserting and twisting until the limb twitched. Finally, the needle penetration depth for PC8, SI3, ST36, SP9, KI3, and LR3 should be between 0.3–2 inches.

##### 2.3.4.4 Retaining needle

The acupuncture manipulation were performed once every 10 min to maintain the needle sensation, a total of 2 times, and the needles were left for 30 min.

##### 2.3.4.5 Needle output

The acupuncture operator uses their left thumb and forefinger to hold a disinfectant dry cotton swab and lightly press it onto the needle site. With their right hand, they hold the needle and twist it slightly before slowly lifting it to the subcutaneous area. Wait for a moment before releasing the needle. After the needle is pulled out, immediately press the needle hole again with a sterilized dry cotton ball to prevent bleeding.

### 2.4 Outcome measures

#### 2.4.1 Primary outcome measures

The Coma Recovery Scale-Revised (CRS-R) can comprehensively assess the consciousness state in terms of auditory, visual, motor, speech, communication, and arousal levels (27). It was also the most sensitive indicator for clinical differentiation of the state of consciousness in patients with pDOC, and has an important reference value in identifying patients with coma, *VS*, MCS, and Emergence from Minimally Conscious State (EMCS), and was therefore considered to be the gold standard for clinical assessment of the level of consciousness (28, 29). Meanwhile, its reliability in monitoring states of consciousness has been commonly recognized. The CRS-R has a full score of 23, and the treatment was considered effective when the CRS-R score at the end of the trial session was greater than or equal to 3 points from the baseline period score (30). Due to the non-constant nature of the state of consciousness of pDOC patients (31), to provide a more reasonable response to the level of consciousness of pDOC patients, we will assess patients’ CRS-R score at 8:00 am, 12:00 pm, and 4:00 pm on 2 consecutive days at baseline and at the end of the experimental observation, respectively, and take the highest value as the data participating in the final statistical analysis.

#### 2.4.2 Secondary outcome measures

The Full Outline of Unresponsiveness Scale (FOUR) is another clinical scale widely used to assess the level of consciousness in patients with pDOC and consists of items such as ocular responses, motor responses, brainstem reflexes, and respiratory status (32). This tool can supplement the CRS-R with assessments such as brainstem reflexes and breathing patterns (33). Also, the higher the score, the better the patient’s level of consciousness. The evaluation time is synchronized with the CRS-R.

The Nociception Coma Scale-Revised (NCS-R) can be used to assess the changes in sensitivity to painful stimuli during treatment in pDOC patients (34), which is a nociceptive behavioral observation tool developed specifically for patients with DOC due to (acquired) brain injury and has good clinical reliability, especially for patients with *VS*/UWS and MCS (35). The scale consists of three items (motor function, verbal function, and facial expression), each with a score range of 0–3. Of note, the presence of pain is indicated when the total NCS-R score is greater than or equal to 4; and the higher the score, the more pronounced the pain response.

The Disability Rating Scale (DRS) can be used to assess the degree of impairment of life functioning in post-traumatic pDOC patients in the domains of awareness (i.e., eye-opening, communication skills, motor responses), cognition (i.e., eating, toileting, and grooming), overall level of functioning, and employability. This study was combined with assessment scales such as CRS-R, FOUR, and NCS-R, which were assessed in several different periods to comprehensively determine the level of consciousness of pDOC patients, aiming to minimize the influence due to fluctuations in the patient’s state of consciousness and the assessor’s subjective experience to ensure the accuracy of the data.

Despite strengths as mentioned above for these standardized clinical scales, it is undeniable that there is always a certain subjectivity in behavioral evaluation. In comparison, the ERP detection technology captures the electrical signals generated by brain activity and has the advantages of high temporal resolution and the ability to assess the information processing capacity of pDOC patients without obvious behavioral manifestations, thus facilitating the discovery of hidden conscious activities in the areas of attention, sensation, emotion, and memory in pDOC patients (36). As a result, we chose the MMN, which reflects the processing of novel events by the brain (37), and P300, an endogenous component that reflects the advanced processing of sensation (38), as the final test indexes.

It has been shown that MMN has a good prognostic value for recovery of consciousness in severely impaired patients (39). For *VS* patients in particular, *VS* patients may have the same ability to be able to process target sound stimuli as MCS patients, suggesting that late-stage DOC patients without motor/behavioral responses still have a cerebral cortex activated by latent stimuli, thus facilitating the discovery of covert conscious activity in pDOC patients (40).

P300 is another ERP testing indicator that correlates with recovery of consciousness and prognosis. It has been suggested that improved awareness may be associated with improved allocation of attentional resources, which can be captured by the P300 amplitude (41). Therefore, in our study, the included pDOC patients will be tested for MMN and P300 at the baseline period and the end of the observation course, respectively, to rationally assess the clinical efficacy of acupuncture-assisted therapy for pDOC.

### 2.5 Sample size

In this trial, CRS-R changes will be regarded as a primary evaluation index. According to our previous pilot study, the changes of CRS-R before and after treatment in the experimental group was shown to be 3.07 ± 2.87 points (*n* = 14), and that in the control group was 1.14 ± 3.80 points (*n* = 14). Hence, sample size calculation was performed using G*Power 3.1.9.7 software (Heinrich-Heine-University Dvsseldorf, Dvsseldorf, Germany) with a significance level (*α* = 0.05) of a 2-sided 2-sample *t*-test and 80% power to detect a difference between the 2 groups. Therefore, a total of 110 participants will be required allowing for 10% of attrition, with 55 cases in each group.

### 2.6 Randomization and allocation concealment

This study was conducted by an independent statistician who generated random numbers by applying Statistical Analysis System (SAS) 9.4 software programming (North Carolina State University, USA). All included pDOC subjects will be randomly assigned to the experimental group and control group. The specific implementation process is as follows: an independent random number administrator is responsible for the management and allocation of all random numbers. For subjects who meet the inclusion criteria and sign informed consent, the researcher in charge of recruitment will first contact the random number administrator by phone or WeChat, and report the basic information of the subjects to him/her before requesting the corresponding random number and grouping, and record them on the subject’s CRF promptly. The randomization number administrator was required to back up the subject’s randomization number, grouping, enrollment time, and basic information. In this trial, the implementation of random grouping and allocation concealment was ensured by the above method.

### 2.7 Blinding

This study was evaluated in a blinded fashion, with a third party who was unaware of the subgroups performing the clinical assessment and efficacy evaluation of all subjects. Blinded statistical analysis was used in the data summarization phase, with triple separation of the investigator, the outcome assessors, and the statistics analyzer of the data.

### 2.8 Informed consent

The subject’s family and (or) legal representative will be informed of the details of this study, including the purpose of the study, potential benefits, and risks, other alternative treatment options available, as well as the rights that the subject has and the obligations that need to be fulfilled. They will also be informed that they will only sign the completed informed consent form before the patient is enrolled in our study. If a subject withdraws from the trial, the relevant assay data will be retained for final analysis of the results.

### 2.9 Safety monitoring

All adverse events occurring during the study period, regardless of whether they are related to acupuncture or not, must be recorded and reported in detail on the corresponding CRF forms of the subjects, mainly including the time of appearance of the adverse events, symptoms, signs, degree, duration, laboratory test indexes, treatments and results, aftermath, follow-up, etc., and analyze the reasons for the appearance of the adverse events. In addition, subjects must be treated promptly and appropriately until they return to normal. The incidence of adverse events will be considered for safety measurements, such as local hematoma, needle twisting, breaking, and bleeding. Also, the investigator is required to keep the subject’s legal representative informed and to ask them to truthfully respond to any changes in the patient’s condition after receiving the treatment. Further, any serious adverse events related to the trial must be reported immediately to the principal investigator, and its cause determined, its relevance to needling analyzed, and an appropriate solution proposed.

### 2.10 Follow-up

Considering that the participants in this study are pDOC patients who are categorized as clinically critical illnesses, they need to receive long-term and continuous clinical treatment as a guarantee to maintain their condition and promote their recovery. In this pilot study, since the observation time of pDOC patients is limited by the duration of hospitalization and patients still need to receive other treatments after discharge, in order to ensure the completeness of the trial implementation, we only followed up the destination of pDOC patients within 1 month of discharge from hospital without assessing or retesting the outcome indexes to ensure the reliability of the data.

### 2.11 Data collection and management

In this study, a CRF specially designed in advance will be applied to collect all the raw data from the subjects and backed up to the corresponding electronic database. Also, there were three examiners in our study, all of whom were trained in a uniform, specialized normative consciousness assessment, and their intraclass correlation coefficient was 0.8, which suggests a high degree of interstudy agreement, thus ensuring the reliability of the measurement data. Furthermore, all subjects who met the criteria for ERP testing were tested by the same one experienced and specialized neurophysiological technologist. Only members of the research team will have access to these data until published in a peer-reviewed journal.

## 3 Statistical analysis

In this study, all collected data will be statistically analyzed by 2 independent professional statisticians applying IBM SPSS Statistics 27.0 software for Windows (Stanford University, USA). Measurement data will be expressed as mean ± standard deviation (¯x ± s) and count data will be expressed as several cases or percentages. Statistical analysis of the measurement data will be done by performing normality and variance chi-square tests, for data that meet the normality and variance chi-square tests, paired samples t-tests will be used for intra-group comparisons, and independent samples t-tests for inter-group comparisons. As for data that do not meet the normality and variance chi-square tests, non-parametric tests will be used. In contrast, the chi-square test will be mainly used for statistical analysis of count data, while the Wilcoxon rank-sum test will be applied when two groups of hierarchical data are compared. For correlation analysis, Pearson’s correlation analysis will be used if the variables conformed to a normal distribution, otherwise Spearman’s correlation analysis will be used. A statistically significant difference or correlation will be indicated when *p* < 0.05. Eventually, all data collected will be analyzed in subgroups by *VS* and MCS populations.

Additionally, in order to evaluate the clinical efficacy and safety of acupuncture-assisted therapy for pDOC patients as comprehensively and rationally as possible, we plan to perform FAS and Per Protocol Set (PPS) analyses, respectively, on all the data eventually collected after comprehensively considering the strengths and weaknesses of the FAS and PPS analysis principles. In terms of the safety evaluation, we will perform statistical analyses based on the Safety Set (SS) principle, aiming to provide study conclusions with a high degree of credibility.

## 4 Quality control

To ensure the reliability of the results, all researchers participating in this trial will receive uniform standardized training and obtain a certificate of competence. To ensure consistency across participants, our group will develop a complete set of clinical management protocols. The above measures can be used to ensure the feasibility and safety of the clinical study.

## 5 Trial status

Patient enrollment for this study began on January 1, 2023, and the study is still ongoing. Protocol version number and date: V3.0, 25/04/2024. Patient enrollment is expected to be completed by October 2024.

## 6 Discussion

PDOC is considered to be a rare neurological disorder (42), and there is a lack of empirical guidance and strong evidence-based medical evidence for clinical diagnosis, treatment, evaluation, and related research. Indeed, it has been demonstrated that the available therapies, such as hyperbaric oxygen, tDCS, transcranial magnetism, and amantadine have limited efficacy to DOC patients (4, 43). In addition, as early as 2000 years ago in the famous ancient Chinese book” The Historical Records,” there are medical records of acupuncture to promote the awakening of DOC. For these reasons, it is promising to improve the clinical wakefulness-promoting efficacy of existing therapies through the combination of acupuncture. Thus, the tDCS and hyperbaric oxygen therapies were included in the conventional treatment of this protocol. Meanwhile, considering the misdiagnosis rate may caused by behavioral evaluation, we selected several standardized clinical scales combined with ERP indicators jointly assess the consciousness level of pDOC patients before and after treatment, aiming to comprehensively evaluate the efficacy of acupuncture-assisted therapy for pDOC patients from the perspective of subjective evaluation combined with objective detection. Additionally, the implementation of this trial will be carried out in strict compliance with the Declaration of Helsinki and Good Clinical Practice Guidelines and will be reported following the Consolidated Standards for Reporting of Trials and the Standards for Reporting Interventions in Controlled Trials of Acupuncture. Meanwhile, for the duration of the pilot study, patients in both groups will continue to receive standard conventional treatment for pDOC to ensure that there is no delay or exacerbation of the patient’s condition as a result of participation in this study.

Nonetheless, some non-negligible limitations should be considered. Firstly, it is undeniable that our study is a single-center study, and there may be limitations in terms of underpowered and poorly generalizable experimental design and implementation, so the final findings should be treated with caution. Secondly, due to the limitation of hospitalization time for pDOC patients in this study, our trial protocol was set up with only 20 acupuncture interventions, which may increase the risk of negative results as a result of short treatment time, but this does not negate the clinical effectiveness of acupuncture-assisted therapy for pDOC. Therefore, in the future, when conditions permit, we should extend the duration of acupuncture treatment as much as possible to improve the credibility of the research results.

Above all, our findings may not only provide useful clinical decision-making options for the rehabilitation of pDOC patients, but also indicate directions for improvement and enhancement in future clinical research.

## Ethics statement

The study was approved by the Ethics Committee of West China Hospital of Sichuan University. In addition, all family members and (or) legal representatives of pDOC patients participating in this trial voluntarily signed an informed consent form, and the results of this trial will be submitted to a peer-reviewed journal for publication.

## Author contributions

NZ: Conceptualization, Software, Writing – original draft. NS: Conceptualization, Software, Writing – original draft. PH: Software, Writing – original draft. L-yY: Software, Writing – original draft. C-xG: Software, Writing – original draft. JX: Software, Writing – original draft. Y-wL: Project administration, Resources, Supervision, Writing – review & editing. HZ: Project administration, Resources, Supervision, Writing – review & editing.
